# Iron Regulation and an Opportunistic AIDS-Related Fungal Infection

**DOI:** 10.1371/journal.pbio.0040427

**Published:** 2006-11-21

**Authors:** Liza Gross

Because HIV attacks the very cells charged with fighting infection, the virus compromises the body’s ability to co-exist with pathogens that are otherwise harmless. It is these pathogen-induced opportunistic infections, and not the virus itself, that produce the most debilitating effects of the disease. The appearance of specific opportunistic infections—including the life-threatening fungal infection cryptococcosis—signals progression to AIDS. Nearly all AIDS-related cryptococcosis cases worldwide are caused by *Cryptococcus neoformans*, a single-celled fungus originally isolated over 100 years ago.

A critical factor in *C. neoformans* infection is iron availability. Because iron also supports fundamental host cell processes, the pathogen must compete with the host to secure enough iron for survival and replication. Genetic and nutritional factors, along with HIV itself, promote iron accumulation in cells and organs, dramatically increasing its availability to *C. neoformans* and other potential pathogens. Understanding how pathogenic fungi sense host resources and control virulence-related factors is essential for developing effective antifungal therapies. In a new study, Won Hee Jung, James Kronstad, and colleagues identify a gene in *C. neoformans* that coordinates both processes, revealing a potentially powerful antifungal strategy. The gene, called *Cryptococcus iron regulator (CIR1)*, regulates not only the pathogen’s response to iron but also its ability to establish virulent infection.

Studies in other fungi, including a harmless laboratory yeast, showed that cell-surface enzymes called reductases facilitate iron uptake by reducing extracellular iron (that is, transforming it into a biologically available state through electron transfer). Such studies also identified three transcription factors involved in maintaining the proper balance, or homeostasis, of cellular iron by repressing the components of the iron-uptake pathway. Using the sequences of these transcription-repressing iron regulators, the authors identified *CIR1* as a candidate regulator in *C. neoformans*. Like the other regulators, the gene contains a region rich in cysteine bases and a “zinc finger motif,” which in the other regulators binds to the promoters of iron transporter genes.

To investigate *CIR1*’s function, the authors deleted its coding sequences from two *C. neoformans* strains. Loss of a transcriptional repressor in the laboratory yeast leads to increased cell-surface reductase activity (which is evident when a colorless indicator dye in the growth medium turns red from the enzyme’s reducing activity). In contrast to the nonmutant, or wild-type, cells, *cir1* mutants appeared reddish. But when the authors added the *CIR1* gene to the mutant cells, they looked the same as the wild-type cells, indicating that the loss of *CIR1* led to increased reductase activity. Mutants also showed signs of sensitivity to excess iron, revealing *CIR1*’s role in iron homeostasis.

**Figure:**
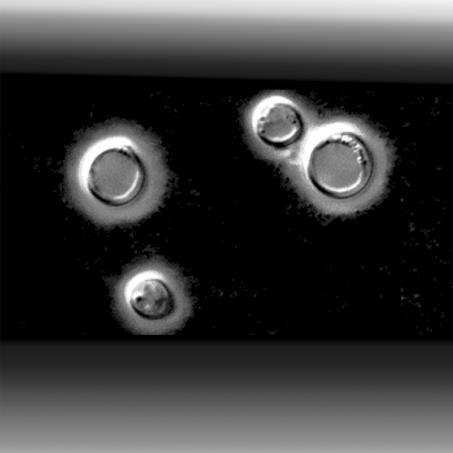
To establish virulent infection, *C. neoformans* must be able to grow at 37 °C, deposit melanin in the cell wall, and produce a polysaccharide capsule (displayed in the image).

To examine how the mutation changed the transcription of iron-related genes, the authors grew mutant and wild-type strains in high and low iron concentrations and then analyzed their gene-expression profiles with microarrays. The profiles of mutant and wild-type strains showed “substantial” differences in both iron backgrounds, indicating that the Cir1 protein senses iron levels and coordinates gene expression accordingly. Genes involved in iron transport were most affected by iron availability and *CIR1* deletion. Based on the microarrays, the authors concluded that Cir1 represses iron uptake mediated by reductases but activates uptake mediated by transport molecules called siderophores. The arrays also revealed that the gene influences melanin production, an important virulence factor that thwarts host antimicrobial proteins.

The authors explored the *cir1* mutation’s effects on *C. neoformans* virulence in the wild-type and mutant strains and found that capsule formation—which disrupts the host’s cellular defenses—was absent in mutant cells. This defect appears to arise in part because the mutation inhibits signaling pathways required for capsule formation. The mutants also showed substantial defects in an absolutely critical virulence factor: the capacity to grow at host body temperature (37 °C). The authors confirmed that *CIR1* exerts significant control over *C. neoformans* virulence in experiments with mice. Mice exposed to a normally virulent strain lacking *CIR1* showed no serious symptoms, while mice infected with strains containing the protein died within 20 days.

Altogether, these findings demonstrate that *CIR1* controls the expression of genes required for *C. neoformans* virulence—and that iron regulation plays a critical role in cryptococcal infection. This intimate connection between iron and virulence suggests that targeting *CIR1* or otherwise disrupting iron regulation might prove an effective strategy for controlling one of the most common life-threatening fungal infections in persons with AIDS.

